# Altered hemodynamics by 4D flow cardiovascular magnetic resonance predict exercise intolerance in repaired coarctation of the aorta: an in vitro study

**DOI:** 10.1186/s12968-021-00796-3

**Published:** 2021-09-06

**Authors:** Jason G. Mandell, Yue-Hin Loke, Paige N. Mass, Vincent Cleveland, Marc Delaney, Justin Opfermann, Seda Aslan, Axel Krieger, Narutoshi Hibino, Laura J. Olivieri

**Affiliations:** 1 Division of Cardiology, Children’s National Hospital, 111 Michigan Ave NW, Washington, DC 20010 USA; 2 Sheikh Zayed Institute for Pediatric Surgical Innovation, Children’s National Hospital, 111 Michigan Ave NW, Washington, DC 20010 USA; 3 Department of Mechanical Engineering, Johns Hopkins University, Latrobe Hall 223, 3400 North Charles St, Baltimore, MD 21218 USA; 4 Section of Cardiac Surgery, Department of Surgery, University of Chicago, 5841 S Maryland Avenue, Chicago, IL 60637 USA; 5 Section of Cardiac Surgery, Department of Surgery, Advocate Children’s Hospital, 4440 West 95th Street, Oak Lawn, IL 60453 USA

**Keywords:** Coarctation of the aorta, 4D flow MRI, Exercise

## Abstract

**Background:**

Coarctation of the aorta (CoA) is associated with decreased exercise capacity despite successful repair. Altered flow patterns have been identified due to abnormal aortic arch geometry. Our previous work demonstrated aorta size mismatch to be associated with exercise intolerance in this population. In this study, we studied aortic flow patterns during simulations of exercise in repaired CoA using 4D flow cardiovascular magnetic resonance (CMR) using aortic replicas connected to an in vitro flow pump and correlated findings with exercise stress test results to identify biomarkers of exercise intolerance.

**Methods:**

Patients with CoA repair were retrospectively analyzed after CMR and exercise stress test. Each aorta was manually segmented and 3D printed. Pressure gradient measurements from ascending aorta (AAo) to descending aorta (DAo) and 4D flow CMR were performed during simulations of rest and exercise using a mock circulatory flow loop. Changes in wall shear stress (WSS) and secondary flow formation (vorticity and helicity) from rest to exercise were quantified, as well as estimated DAo Reynolds number. Parameters were correlated with percent predicted peak oxygen consumption (VO2_max_) and aorta size mismatch (D_AAo_/D_DAo_).

**Results:**

Fifteen patients were identified (VO2_max_ 47 to 126% predicted). Pressure gradient did not correlate with VO2_max_ at rest or exercise. VO2_max_ correlated positively with the change in peak vorticity (R = 0.55, p = 0.03), peak helicity (R = 0.54, p = 0.04), peak WSS in the AAo (R = 0.68, p = 0.005) and negatively with peak WSS in the DAo (R = − 0.57, p = 0.03) from rest to exercise. D_AAo_/D_DAo_ correlated strongly with change in vorticity (R = − 0.38, p = 0.01), helicity (R = − 0.66, p = 0.007), and WSS in the AAo (R = − 0.73, p = 0.002) and DAo (R = 0.58, p = 0.02). Estimated DAo Reynolds number negatively correlated with VO2_max_ for exercise (R = − 0.59, p = 0.02), but not rest (R = − 0.28, p = 0.31). Visualization of streamline patterns demonstrated more secondary flow formation in aortic arches with better exercise capacity, larger DAo, and lower Reynolds number.

**Conclusions:**

There are important associations between secondary flow characteristics and exercise capacity in repaired CoA that are not captured by traditional pressure gradient, likely due to increased turbulence and inefficient flow. These 4D flow CMR parameters are a target of investigation to identify optimal aortic arch geometry and improve long term clinical outcomes after CoA repair.

## Background

Coarctation of the aorta (CoA) is associated with decreased exercise capacity despite successful repair [1, 2], though the hemodynamic mechanism remains unknown. Current guidelines focus on significant aortic isthmus narrowing as a target of re-intervention [3, 4] with more recent studies considering intervention for increased pressure gradient uncovered by pharmacologic stress test during cardiac catheterization [5, 6]. Previous work suggests aortic arch shape after CoA repair, even in the absence of re-coarctation, is associated with important clinical outcomes, such as left ventricular (LV) function [7] and late systemic hypertension [8]. Our recent study identified aorta size mismatch, defined by the ascending aorta (AAo) to descending aorta (DAo) diameter ratio, D_AAo_/D_DAo_, as an important indicator of decreased exercise capacity after CoA repair [9].

Changes in arch geometry likely increase resistance to flow [10], altering flow characteristics which can be measured by 4D flow cardiovascular magnetic resonance (CMR) (time-resolved 3D phase-contrast CMR with 3D velocity encoding) [11]. Increased wall shear stress (WSS), the component of force parallel to the vessel wall [12, 13], is an important cause of aortopathy [14] and is altered after CoA repair at both rest [15, 16] and exercise [17]. Additionally, 4D flow CMR can quantitatively assess secondary flow formation described by vorticial and helical patterns, which are normal physiologic flow features in the thoracic aorta [18–20] and are affected by changes in arch geometry [21–24]. Vorticity is defined by the curl of the velocity field, i.e. the vector described by the magnitude of local rotation about an axis of flow, multiplied by the velocity vector. Vorticity is therefore proportional to both organized rotation and velocity [21, 25]. Helicity is a scalar defined by the dot product of the local vorticity and velocity vectors and describes the rotation of the vortex field creating a corkscrew pattern [20, 21, 25]. Vorticity and helicity in the pulmonary arteries of patients with pulmonary artery hypertension were described as markers of right ventricular (RV) afterload and function [25, 26], though effects have yet to be described specific to the LV.

Altered aortic arch geometry likely impacts the development of turbulent flow, especially in states of increased cardiac output such as exercise. The Reynolds number describes the transition from laminar to turbulent flow with a lower number predicting laminar flow and a higher number predicting turbulent flow. It is proportional to the flow rate and inversely proportional to fluid viscosity and vessel diameter [27]. The Reynolds number has been used to estimate turbulent flow in normal aortas and a critical peak Reynolds number, i.e. transition point to turbulent flow, was estimated to be approximately 4000 in the DAo [28], though is dependent on exact geometry and will be different for each aorta. As WSS is also proportional to velocity [12], it may serve as a marker of local turbulent flow. Helical and vorticial flow formation in the aorta has been proposed as a mechanism to provide flow stability and reduce turbulence [25, 29]. These secondary flow characteristics may be indicators of the degree of flow efficiency (i.e. laminar vs turbulent flow), and therefore deserve investigation as biomarkers of exercise intolerance in repaired CoA.

There has been limited evaluation of changes in secondary flow characteristics with exercise, as measured by 4D flow CMR, and their relationship to clinical exercise capacity. We hypothesized that decreased exercise capacity after CoA repair is related to inefficient flow secondary to changes in aortic arch geometry. We used 4D flow CMR to quantify WSS, vorticity, helicity, and Reynolds number during simulations of rest and exercise with three-dimensional printed aortas in an in vitro mock circulatory flow loop.

## Methods

### Subjects

This retrospective study was approved by the Children’s National Hospital Institutional Review Board (number Pro00010748, approved August 24, 2018). Patients with repaired CoA were included if they had a CMR study and an exercise stress test within one year measuring peak oxygen consumption (VO2_max_) and peak respiratory exchange ratio ≥ 1.1, indicating maximal effort. Subjects were excluded with recurrent CoA requiring intervention or if additional medical morbidities were present, such as complex congenital heart disease (i.e. single ventricle), significant valvar regurgitation or stenosis, or significant pulmonary, musculoskeletal, or metabolic problems that affect exercise capacity. The presence of a bicuspid aortic valve was not an exclusion criterion given the high prevalence in this population. Exercise capacity was defined as percent predicted VO2_max_ for age and sex. Per clinical protocol, predicted VO2_max_ was calculated using the James equation [30] for pediatric patients (ages 7–15 years) and the Wasserman equation [31] for adult patients (ages 16–54 years).

### In vivo imaging

All clinical CMR imaging was performed to the lab standard and consistent with Society for Cardiovascular Magnetic Resonance congenital heart disease guidelines [32] on a 1.5 T CMR system (Aera, Siemens Healthineers, Erlangen, Germany) including 3D steady state free precession and angiography with typical parameters to achieve a slice thickness of 1.2–1.5 mm and pixel resolution between 1.2 × 1.2 mm and 1.5 × 1.5 mm, as well as phase contrast imaging and cine volumetry.

AAo and DAo diameters were measured using 3D data sequences and flow was calculated from 2D phase contrast imaging using standard techniques (Medis, Leiden, The Netherlands). Percent DAo flow was calculated to calibrate the in vitro model. Where available, LV ejection fraction (LVEF) and LV mass were obtained from the clinical CMR cine imaging.

The echocardiogram most recent to the exercise stress test was reviewed. LVEF or fractional shortening was measured if the LVEF was not measured by CMR. Additionally, peak DAo velocity, corrected for increased proximal velocity (> 2 m/s), was assessed.

### In vitro model

The details of the CMR-compatible mock circulatory flow loop were previously described [9]. Briefly, a patient-specific three-dimensional model was created using standard segmentation techniques in commercial software (Materialise Mimics/3-matic, Leuven, Belgium). In the cases of stents, there was assumed to be no in-stent stenosis based on review of peak velocity by echocardiogram and dark blood CMR. Therefore, the inner diameter of the vessel was segmented as the same diameter as the proximal and distal native vessel. Models were printed in a rigid plastic (Accura 60; 3D Systems Corporation, Rock Hill, South Carolina, USA) by an additive manufacturing company (Xometry, Gaithersburg, Maryland, USA). To simulate the effect of the aortic shape on hemodynamics, both during rest and exercise conditions, the aorta models and flow parameters were scaled to accommodate the operating limits of the flow pump (CardioFlow 5000MR; Shelley Medical Imaging Technologies, London, Ontario, Canada), requiring a peak instantaneous flow rate less than 300 mL/s. Dimensional analysis, a standard engineering technique, was used to scale flow by decreasing fluid viscosity and arch size while maintaining the same flow conditions [33]. Viscosity was decreased by using water instead of blood-like fluid. The models were scaled linearly by each axis to a body surface area (BSA) of 1 m^2^ based on defined normal values of aorta size in the range of adolescent and adult BSA [34]. By scaling to the same body surface area (BSA), the indexed flow conditions of rest and exercise remained identical for all models (3 L/min/m^2^ and 9 L/min/m^2^, respectively). Flow curves were derived from the AAo flow profile from in vivo CMR.

The printed models were placed in a mock circulatory flow loop. The aorta model was placed in a box filled with water and saline bags for stabilization. The head vessels were combined as a single outflow. Compliance and resistance components were added using standard techniques [12], mimicking in vivo vessel compliance and systemic vascular resistance. The outlet valves were adjusted until the desired flow distribution among head vessels and DAo matched in vivo CMR conditions and remained unchanged between rest and exercise conditions. Therefore, the only the change between the rest and exercise condition was in increase in flow from 3 to 9 L/min/m^2^.

### Pressure measurements

Pressure was measured at access points in the proximal AAo and distal DAo using MR-conditional pressure transducers (Utah Medical Products, Midvale, Utah, USA) with data acquisition via LabView (National Instruments, Austin, Texas, USA). The peak pressure gradient (ΔP) was calculated for rest and exercise conditions by subtracting the DAo peak pressure from the AAo peak pressure averaged over at least ten cardiac cycles.

### In vitro* CMR*

A standard amount of gadolinium contrast was added to the flow circuit and the CMR-compatible mock circulatory flow loop was centered in the bore of a 1.5 T CMR system (Aera, Siemens Healthineers). Acquisitions were gated to the flow pump using the above CMR-conditional pressure sensors [35]. An in-plane 2D phase contrast sequence was used to determine the appropriate encoding velocity to avoid aliasing for exercise flow. 4D flow acquisitions were performed using encoding velocity 200 cm/s for rest and 200–400 cm/s for exercise, echo time 2.2 ms, repetition time 38 ms, flip angle 15 degrees. The acquired matrix size was approximately 72 × 160 with a field of view of 190 mm × 270 mm to obtain a reconstructed resolution of 1.7 mm × 1.7 mm with a slice thickness of 1.8 mm. 4D flow sequences were exported for off-line analysis.

### 4D flow analysis

Each dataset was segmented to isolate the velocity flow fields within the region of interest. To obtain consistent results, a semi-automated threshold technique was developed based on a modified Otsu method [36] using the logarithm of the histogram to avoid bias toward the larger background region [37]. Further post-processing was performed using iTFlow, commercially available software (Cardio Flow Design, Tokyo, Japan), for qualitative assessment of streamline flow and quantitative assessment of peak systolic WSS, vorticity, and helicity. Peak WSS was measured in the AAo, aortic arch, and DAo, defined by standard landmarks [38]. Peak vorticity, and right (positive) and left-handed (negative) helicity were measured in the entire aorta model and normalized by the volume of the segmented region as they are all dependent on voxel volume. Each parameter was measured in rest and exercise conditions, and the ratio of the exercise to rest condition was calculated. Background correction was not applied as there was no static tissue in the in vitro setup to be used for correction. Flow measurements from 4D flow were validated using a clamp-on ultrasonic flow meter (PXL Flowsensor; Transonic, Ithaca, New York, USA). In order to test the hypothesis that increased turbulence in the DAo is related to exercise capacity, the Reynolds number in the DAo (Re_DAo_) was estimated using the average diameter in the DAo (by centerline analysis) and the flow at peak systole in the DAo during rest and exercise, both measured with iTFlow.

### Statistical methods

All statistical analyses were performed with Prism 8 (Graphpad, San Diego, California, USA). A paired two-tailed t-test was used to evaluate the mean difference of ΔP at rest and exercise. All correlations were performed using Pearson’s correlation coefficient (r) including ΔP at rest and exercise with percent predicted VO2_max_, the ratio of exercise to rest for each 4D flow parameter (peak WSS, vorticity, and helicity) with D_AAo_/D_DAo_ and percent predicted VO2_max_, and the estimated Re_DAo_ at rest and exercise with percent predicted VO2_max_.

## Results

### Demographics

Fifteen patients (12–41 years, mean 26.8 ± 8.6 years) met inclusion criteria, the same population as our previous work [9]. Exercise stress testing was performed using the Bruce protocol on a treadmill for all patients except one, who used the 10 W bicycle ergometry protocol. VO2_max_ ranged from severely decreased to normal and was mildly decreased on average (47–126% predicted, mean 92%), consistent with previous reports [1]. Primary repair was done by end-to-end anastomosis (n = 5), long segment patch (n = 4), subclavian flap (n = 4), and stent angioplasty (n = 1), with one unknown surgical repair. Approximately half of the patients had the primary repair at < 1 year of age (n = 8) with the rest occurring at age 1–9 years (n = 6), and one patient at 18 years. Six patients required a secondary repair which was done by balloon angioplasty (n = 5), stent angioplasty (n = 1), or interposition graft (n = 1). Five patients (33%) had hypertension and three patients (20%) were being treated with medication. The full details of their clinical data were previously published [9] and are summarized in Table 1.

**Table 1:** Demographics and clinical data (15 patients)

	Mean ± SD	Range
Age (years)	26.8 ± 8.6	12–41
Years between primary repair and CMR (n = 15)	23.9 ± 8.9	12–39
Years between secondary repair and CMR (n = 7)	15.9 ± 5.8	4–21
Days between CMR and stress test	22 ± 41	0–153
VO2_max_ (% predicted)	92 ± 20	47–126
LVEF	67 ± 7	57–79
D_AAo_/D_DAo_	1.4 ± 0.4	0.9–2.4
Residual peak velocity (m/s)	1.8 ± 0.4	1.1–2.4

*AAo* Ascending aorta, *DAo* descending aorta, *CMR* cardiovascular magnetic resonance, *LVEF* left ventricular ejection fraction, *VO2max* maximum oxygen consumption

### Pressure measurements

**Fig. 1:**
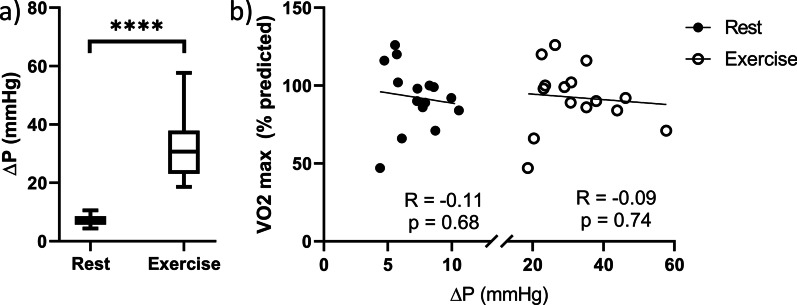
Pressure gradient (ΔP) at rest and exercise with correlations to exercise capacity. **a** Mean ΔP at rest and exercise are significantly different (p < 0.001) shown with line at mean value, box boundaries at 25th and 75th percentiles, and whiskers at minimum and maximum values. **b** ΔP did not correlate with VO2 max at rest or exercise (R = − 0.11, p = 0.68; R = − 0.09, p = 0.74; respectively)

ΔP increased significantly from rest to exercise (Fig. 1, Table 2). There was no clinically significant pressure gradient across the arch at rest (mean 7 mmHg, range 4–11 mmHg). During exercise, ΔP increased significantly (p < 0.001; mean 32 mmHg, range 19–58 mmHg). ΔP did not correlate with VO2_max_ at rest or exercise (Fig. 1).

**Table 2:** Measured values at simulated rest and exercise

	Rest	Exercise	p-value
ΔP (mmHg)	7.2 ± 1.8	32.1 ± 10.9	< 0.0001
AAo peak WSS (Pa)	5.02 ± 1.11	13.34 ± 3.32	< 0.0001
Arch peak WSS (Pa)	6.67 ± 1.27	19.09 ± 3.14	< 0.0001
DAo peak WSS (Pa)	7.84 ± 1.52	23.15 ± 4.53	< 0.0001
Peak vorticity (s^−1^)	103.4 ± 27.4	261.3 ± 82.3	< 0.0001
Peak right helicity (m/s^2^)	3.84 ± 6.10	49.90 ± 79.10	0.0295
Peak left helicity (m/s^2^)	− 13.57 ± 10.94	− 108 ± 87.65	0.0003
Re_DAo_	2995 ± 508	8957 ± 1419	< 0.0001

Values for all investigated parameters at rest and exercise. Comparison of rest to exercise was tested for significance using an unpaired two-tailed t-test. Aortic arch [arch]; ascending aorta (AAo); descending aorta (DAo); pressure gradient (ΔP); Reynolds number in descending aorta (Re_DAo_); wall shear stress (WSS)

### WSS measurements

**Fig. 2:**
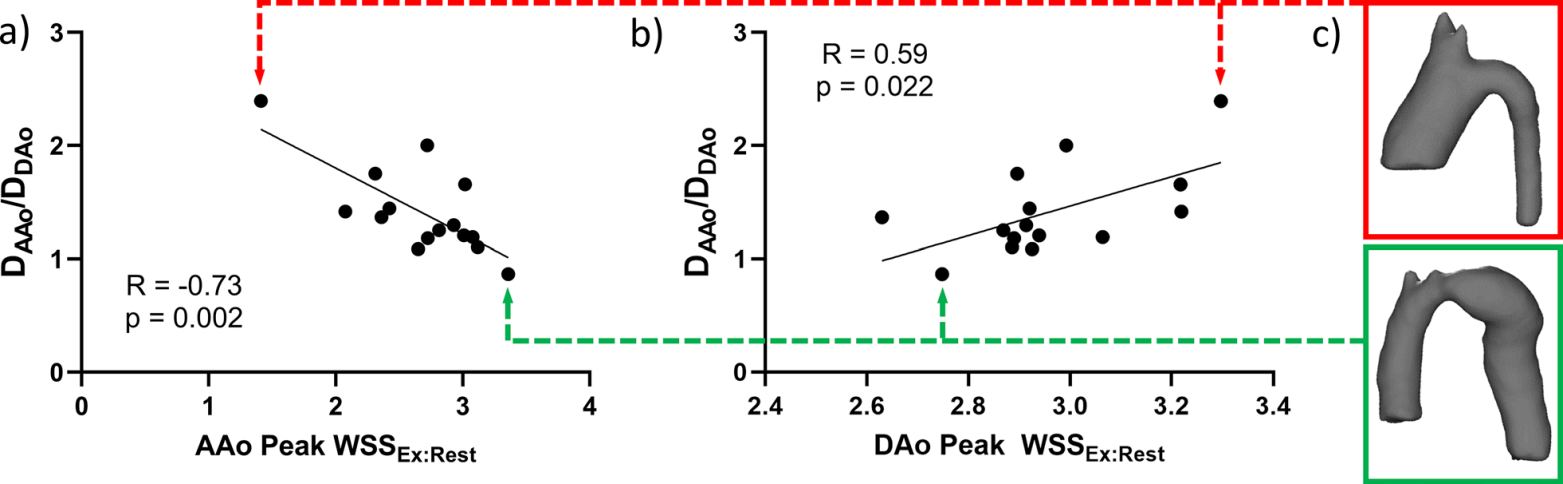
Correlations of the ratio of peak wall shear stress (WSS) in exercise to rest (WSS_Ex:Rest_) in the ascending aorta (AAo) and descending aorta (DAo) with aorta size mismatch (D_AAo_/D_DAo_). **a** AAo peak WSS_Ex:Rest_ had a negative correlation with D_AAo_/D_DAo_. **b** DAo peak WSS_Ex:Rest_ had positive correlation with D_AAo_/D_DAo_. **c** Examples are shown of an aorta with a large D_AAo_/D_DAo_ (red box) leading to a small change in peak WSS in the AAo and a large change in the DAo, while an aorta with a more normal, small size mismatch (green box) leads to a large change in peak WSS in the AAo and small change in the DAo

**Fig. 3:**
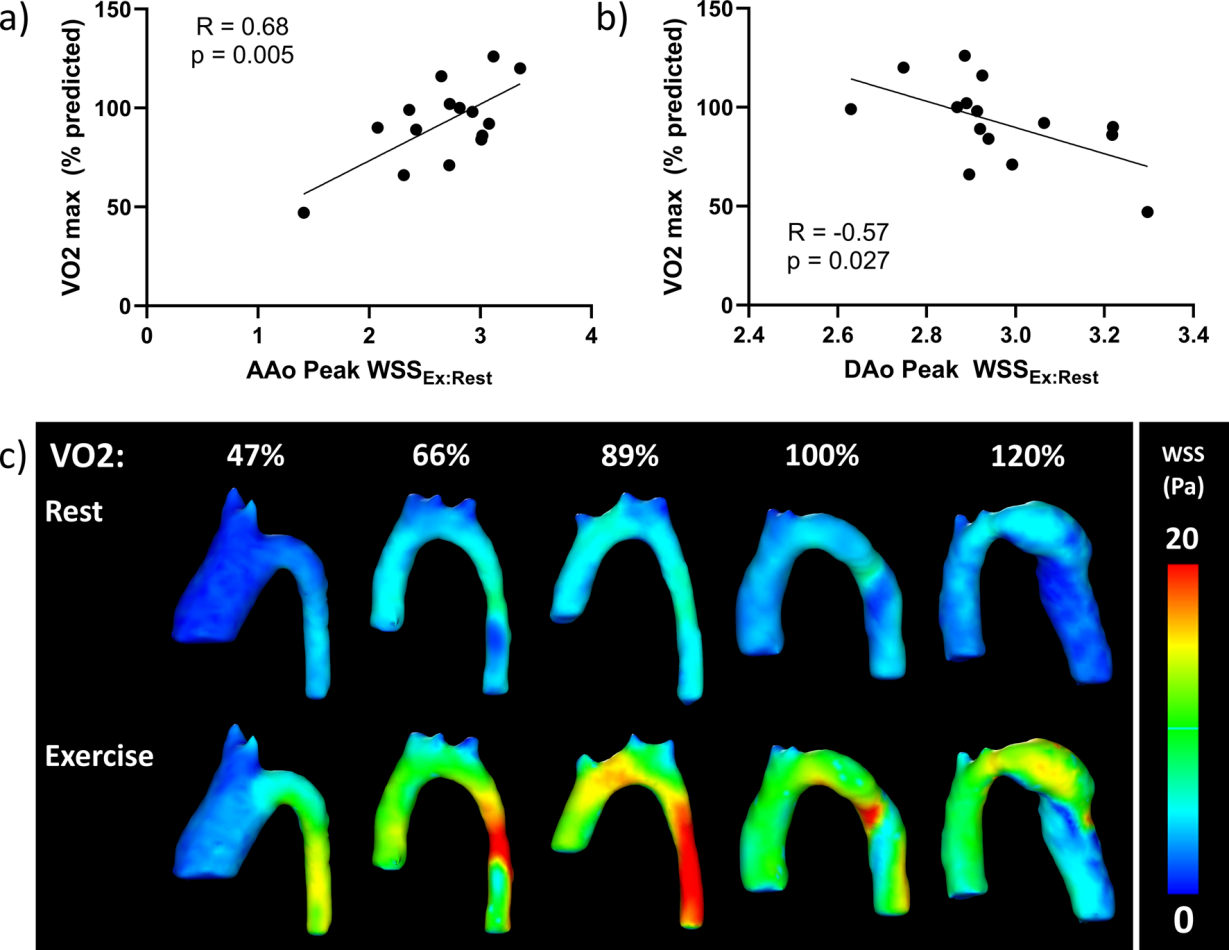
The relationship of the ratio of peak WSS in exercise to rest (WSS_Ex:Rest_) in the ascending aorta (AAo) and descending aorta (DAo) with exercise capacity (VO2 max). **a** AAo Peak WSS_Ex:Rest_ correlated positively with VO2 max. **b** DAo Peak WSS_Ex:Rest_ correlated negatively with VO2 max. **c** Representative peak WSS patterns in aortas of patients in order of VO2 max during simulated rest and exercise. With worse VO2 max, there is a lower change of peak WSS seen in the moderately dilated AAo while there is a larger change in WSS in the relatively small DAo. With better VO2 max, there is a higher change of WSS from rest to exercise in the normal size AAo while lower change in peak WSS is seen in the mildly dilated DAo

Peak WSS increased significantly from rest to exercise in all locations (Table 2). The change in AAo peak WSS from rest to exercise correlated negatively with D_AAo_/D_DAo_ (R = -0.73, p = 0.002), while change in DAo peak WSS correlated positively with D_AAo_/D_DAo_ (R = 0.59, p = 0.022) (Fig. 2). Since WSS is directly related to velocity and velocity increases exponentially with increased flow in the same caliber vessel, it is expected that a smaller D_AAo_/D_DAo_ leads to larger change in AAo peak WSS. Likewise, as D_DAo_ is the denominator of aorta size mismatch, the relationship with WSS is inverse and a smaller D_AAo_/D_DAo_ leads to a smaller change in DAo peak WSS in the setting of increased cardiac output. This is demonstrated in the models with normal exercise capacity in Fig. 3c.

The change in AAo peak WSS from rest to exercise correlated positively with % predicted VO2_max_ (R = 0.68, p = 0.005), while change in DAo peak WSS from rest to exercise correlated negatively with % predicted VO2_max_ (R = − 0.57, p = 0.027) (Fig. 3a, b). There was no significant correlation of the change in peak WSS in the arch with VO2 (R = 0.03, p = 0.91) or D_AAo_/D_DAo_ (R = 0.15, p = 0.58). Visualization of peak WSS in the entire aorta is shown, demonstrating that better exercise capacity is associated with 1) a smaller, less dilated AAo leading to a larger change in WSS, and 2) a larger, more normal DAo leading to a smaller change in WSS (Fig. 3c).

### Vorticity and helicity measurements

**Fig. 4:**
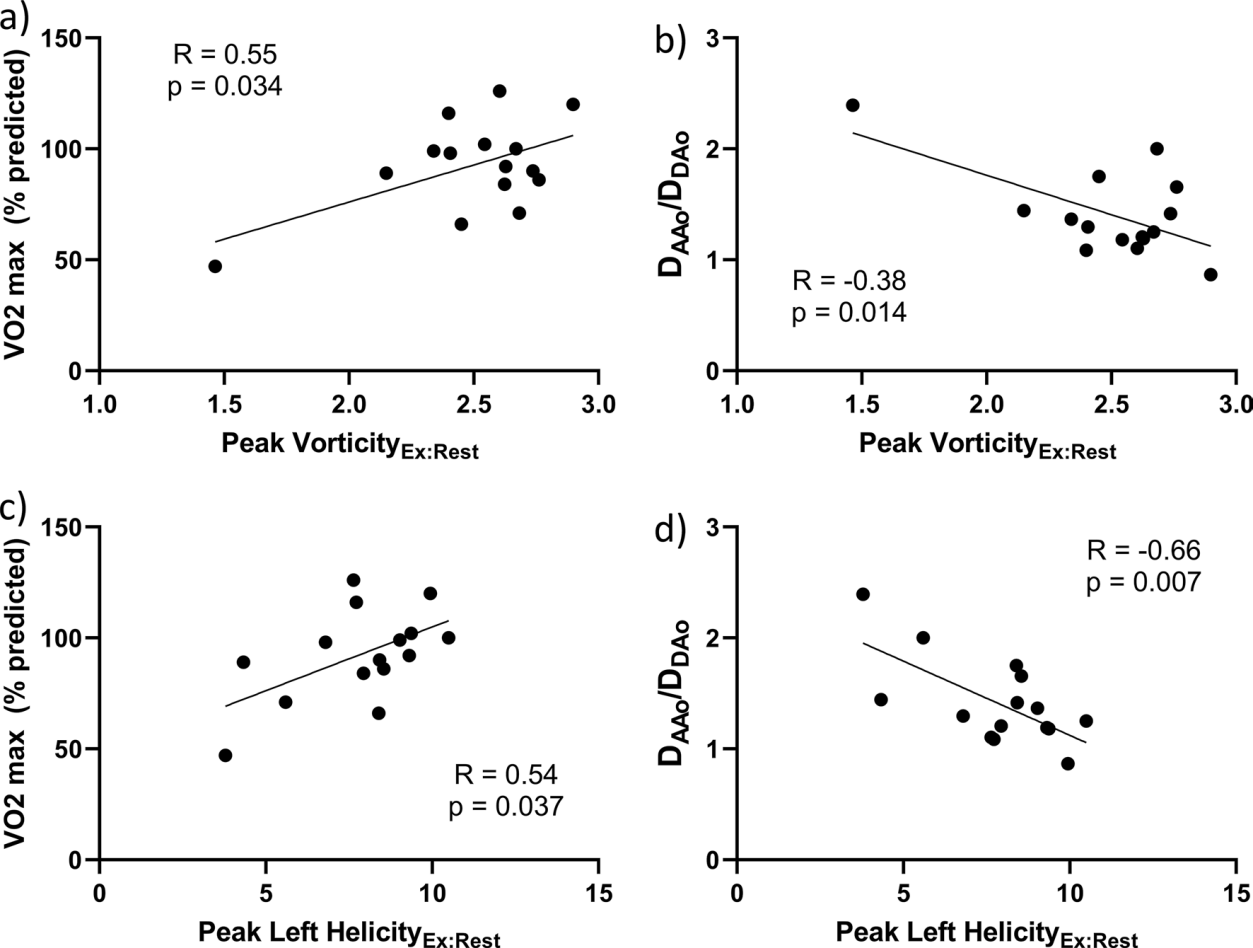
Correlations of the ratio of peak vorticity and peak left helicity in exercise to rest with aorta size mismatch (D_AAo_/D_DAo_) and exercise capacity (VO2 max). **a** Peak vorticity_Ex:Rest_ correlated positively with VO2 max and **b** negatively with D_AAo_/D_DAo_. **c** Similarly, peak left helicity_Ex:Rest﻿_ correlated positively with VO2 max and **d** negatively with D_AAo_/D_DAo_

**Fig. 5:**
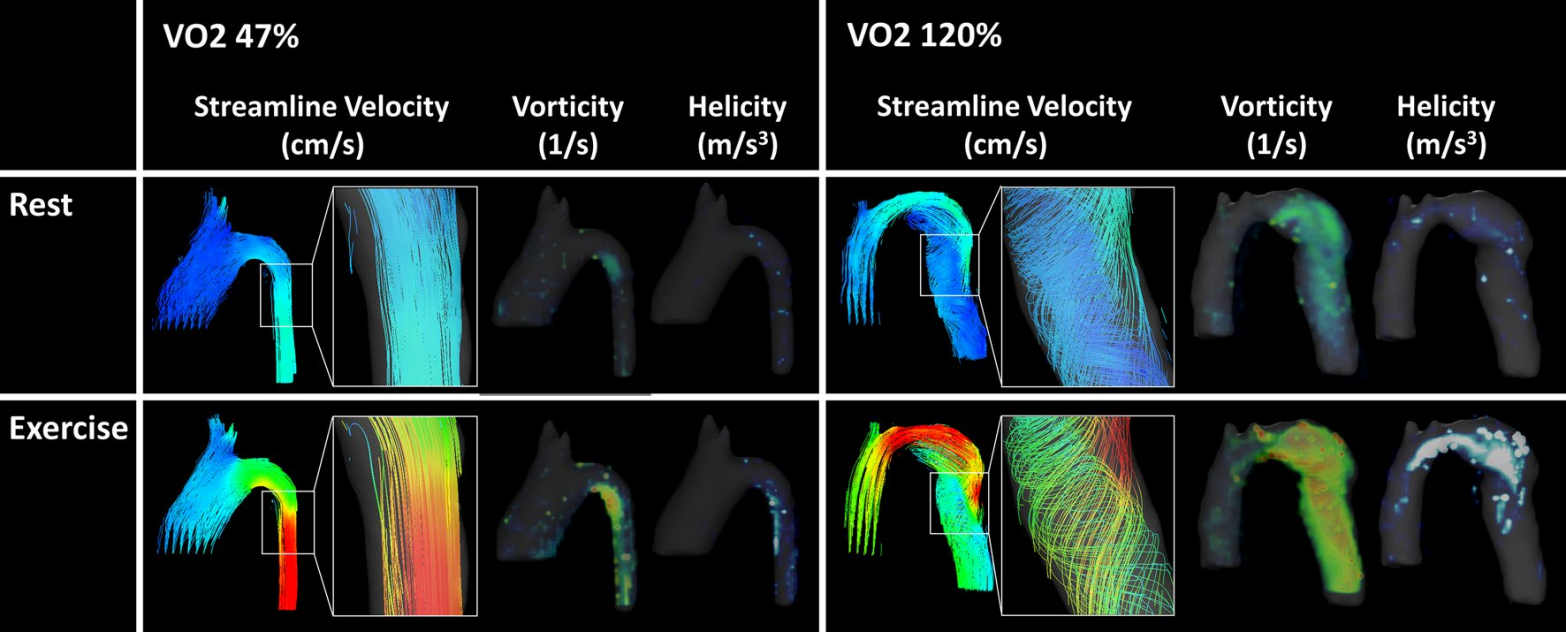
Representative aortas of patients with poor (left) and excellent (right) exercise capacity (VO2 max) during simulated rest (top) and exercise (bottom). Visualizations of streamline flow, vorticity, and helicity are shown for each. More complex secondary flow formation and increased changes in vorticity and helicity are seen with better VO2 max

Peak vorticity and helicity of the whole aorta increased significantly from rest to exercise (Table 2). The change in peak vorticity from rest to exercise positively correlated with % predicted VO2_max_ (R = 0.55, p = 0.034), and negatively with aorta size mismatch (R = -0.38, p = 0.014). Similarly, the change in peak left helicity from rest to exercise positively correlated with % predicted VO2_max_ (R = 0.54, p = 0.037), and negatively with aorta size mismatch (R = -0.66, p = 0.007) (Fig. 4). Change in peak right helicity did not correlate significantly with % predicted VO2_max_ or aorta size mismatch. Streamline visualizations demonstrate increased secondary flow pattern (vorticity and helicity) formation in the larger DAo of the patient with better exercise capacity (Fig. 5).

### Reynolds number analysis

**Fig. 6:**
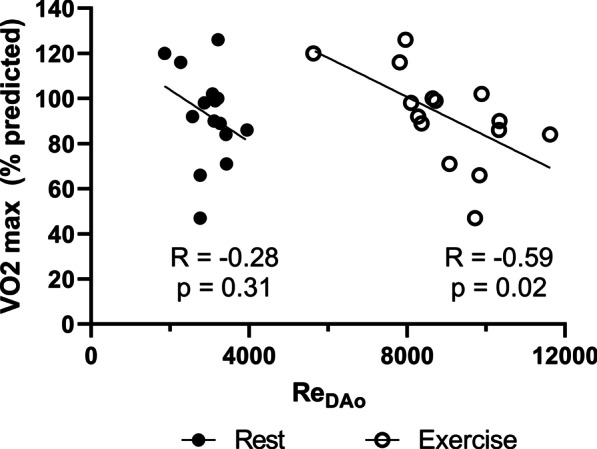
Percent predicted VO2 max versus estimated Reynolds number in the descending aorta (Re_DAo_) for rest and exercise. Re_DAo_ in exercise has a significant negative correlation with VO2. The correlation of Re_DAo_ at rest with VO2 max did not reach significance

**Fig. 7:**
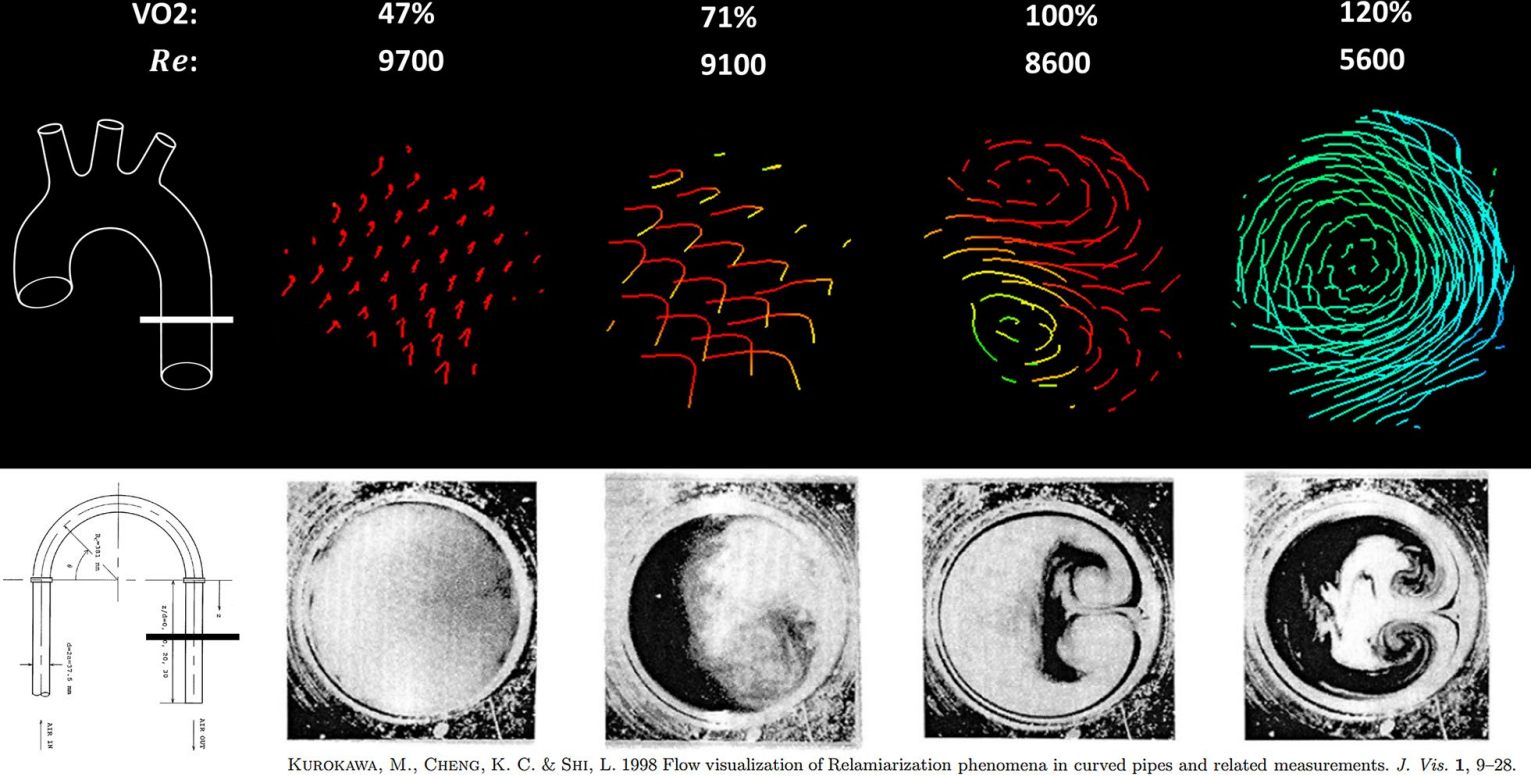
Visualization of flow by 4D flow CMR compared to smoke injection in a curved pipe. Top) Streamline flow by 4D flow CMR in a cross section of the distal descending aorta (DAo) as shown in the schematic on the left in four example patients of varying exercise capacity (VO2 max) from poor to excellent (left to right). Increasing secondary flow formation is seen as Reynolds number (Re) decreases and VO2 increases. Bottom) Reproduced and adapted from Kurokawa et al (CC BY license 4.0): cross-sections of pipe after a 180 degree bend (location shown at left) with decreasing Re from left to right. High Re leads to completely turbulent flow with no secondary flow visualization (left). With decreasing Re, secondary flow formation strengthens creating fully developed Dean vortices at low Re (right)

Re_DAo_ increased significantly from rest to exercise (Table 2). During rest, Re_DAo_ did not correlate significantly with VO2_max_, however, there was a significant negative correlation at exercise (R = − 0.59, p = 0.02) with an increased Re_DAo_ corresponding to decreased exercise capacity (Fig. 6). A cross-section of the streamline flow formation in the DAo is shown in Fig. 7, demonstrating increasing secondary flow formation with decreasing Reynolds number. Additionally, the formation of Dean vortices, or two counter-rotating secondary flow structures [15, 39], is demonstrated in one model with normal exercise capacity.

## Discussion

This study identified several important parameters by 4D flow CMR with in vitro simulations of rest and exercise that are dependent on aortic arch geometry and not captured by pressure gradient alone. Changes in these flow patterns with increased cardiac output are directly correlated with exercise capacity after CoA repair in the absence of significant arch obstruction. Specifically, our model demonstrated that increased turbulence in the DAo with exercise predicts decreased exercise capacity in this population.

Simulations of rest demonstrated no clinically significant pressure gradient across the arch, consistent with in vivo data. With the increased flow of the exercise condition, the pressure gradient increased as expected. Arch obstruction uncovered by exercise has been proposed as a mechanism of exercise intolerance and a target of re-intervention [5]. However, exercise intolerance remains even without arch obstruction, and in this study, pressure gradient with rest or exercise did not correlate with exercise capacity.

Measurements of WSS demonstrated that increased change in peak WSS in the AAo was associated with better exercise capacity, and was seen in patients with a smaller, less dilated AAo. Since our model maintained the same inflow across models, and WSS is linearly dependent on peak velocity [12], WSS in the AAo is exponentially dependent on vessel diameter. It follows that a smaller AAo leads to increased WSS which would increase nonlinearly with a smaller vessel diameter. Decreased change in peak WSS in the DAo was associated with better exercise capacity and was seen in patients with a larger, more normal DAo. Similarly, WSS in the DAo is dependent on diameter, though it is also dependent on the amount of flow in the DAo. These geometrical changes are captured by aorta size mismatch. Better exercise capacity is associated with a smaller D_AAo_ and larger D_DAo_, thus overall smaller D_AAo_/D_DAo_. Although, a smaller D_AAo_/D_DAo_ leads to more DAo flow [9], velocity is linearly dependent on flow compared to the square of the vessel diameter, making diameter a more important contributor to WSS. These changes in WSS are more likely secondary to arch geometry rather than a cause of exercise intolerance. However, abnormal WSS remains clinically important, likely leading to vessel remodeling and altered vascular and endothelial function [14, 40]. Notably, there was no significant correlation of the change in WSS in the aortic arch with exercise capacity or aorta size mismatch.

Analysis of secondary flow formation demonstrated better exercise capacity to be associated with increased changes of peak vorticity and helicity. Each parameter was also negatively correlated with aorta size mismatch. Because the in vitro model was designed to assess the aorta and not the LV outflow tract, inflow into the AAo was fully developed, differing from an in vivo model which may be affected by the presence of a bicuspid aortic valve. It was therefore expected that changes in secondary flow patterns were more apparent in the arch and DAo (Fig. 5). As there is a relative dominance of left helicity in the DAo of a left aortic arch [19], this may explain why we found a significant correlation of peak left helicity, and not right helicity with exercise capacity in our population of only left arches. There remains the possibility that right helicity could be an important determinant of exercise capacity in the setting of an abnormal LV outflow tract. We found that vorticity and helicity increased more in patients with a larger DAo. Our findings are consistent with previous studies, suggesting that increased vorticial and helical flow formation are associated with more efficient and less turbulent flow, emphasizing that quantitative vorticity and helicity are different than qualitative large-scale flow formation. Unlike qualitative measures, quantitative vorticity and helicity are proportional to velocity and cohesiveness of flow, leading to an increase with stable, laminar flow [25, 29].

Subsequent evaluation demonstrated that a higher Re_DAo_ at exercise was associated with decreased exercise capacity. The higher Reynolds number indicates there is more turbulent flow in the DAo despite the straight appearance of the streamlines (Fig. 7). These findings are reminiscent of a previous study by Kurokawa et al. evaluating secondary flow formation in a curved pipe with smoke injection. They found that in the case of a 180-degree bend, secondary flow was seen at low Reynolds number. As Reynolds number increased, the visualized flow formation disappeared due to turbulent flow causing smoke diffusion. Thus, any existing secondary flow was under the limits of detection given the spatial and temporal resolution of their methods [39]. Similarly, it is likely that increased turbulence results in decreased secondary flow formation as measured by conventional 4D flow CMR which averages velocities in each voxel over time. More advanced methods may be required to fully capture this phenomenon, such as turbulent kinetic energy which measures intravoxel velocity fluctuations [41, 42].

### Limitations

There are several important limitations of this study to consider. This study evaluated a heterogeneous cohort with different types of repair at different ages resulting in a variety of aortic arch shapes. With regards to the in vitro model, the aorta models were printed in a hard polymer. The compliance and resistance components model vascular compliance, though they do not account for vascular dysfunction or changes in compliance due to primary aortopathy or post-operative changes such stents or patches. Additionally, our exercise model keeps compliance and resistance elements unchanged from rest to exercise which does not account for changes in systemic vascular resistance and subsequent changes in flow distribution. We suspect that if there is a disproportionate decrease in the systemic vascular resistance of the lower extremities with exercise, there will be a further increase in DAo flow, and would amplify our findings related to turbulent DAo flow. Further work will be needed to understand the limitations of spatial and temporal resolution in the measurements of secondary flow characteristics. Despite these limitations, the in vitro flow pump is ideal for isolating the contribution of arch geometry to changes in flow and this work serves as an important first step towards understanding how post-operative arch shape impacts exercise capacity.

This study has important implications for the applications of in vivo 4D flow that are worth further investigation. First, 4D flow CMR measurements of turbulence may be able to better predict exercise intolerance compared to simplified 1-dimensional measurements of the aorta, especially in cases of complex arch shapes. Second, as 3D-printed surgical grafts become a reality, 4D flow parameters can be used to design a more efficient arch that is personalized for each patient. To realize these goals, it will be necessary to investigate these 4D flow parameters in vivo at rest, and potentially during exercise. Additionally, evaluation of turbulent kinetic energy during exercise will likely be an important indicator of flow efficiency. Statistical shape modeling can be used to better understand favorable geometric changes. Further work is needed to determine how surgical technique influences long term arch geometry compared to non-modifiable factors such as intrinsic shape and remodeling secondary to vasculopathy. Finally, computational fluid dynamics can be used to model changes in arch shape with “virtual surgery.” Together, these methods have the potential to establish goals of re-intervention beyond reduced pressure gradient.

## Conclusion

This study identified important associations between secondary flow characteristics and exercise capacity in repaired CoA not captured by traditional pressure gradient, likely due to increased turbulence and inefficient flow. Quantitative 4D flow CMR analysis holds strong potential to identify optimal aortic arch geometry, establish new goals of re-intervention, and improve long term clinical outcomes after CoA repair.

## Data Availability

The datasets generated and analyzed during the current study are not publicly available in order to protect subject anonymity, though are available from the corresponding author on request.

## Abbreviations

ΔP: Pressure gradient
4D: Four-dimensional
4D flow CMR: Time-resolved 3D phase-contrast CMR with 3D velocity encoding
AAo: Ascending aorta
BSA: Body surface area
CMR: Cardiovascular magnetic resonance
CoA: Coarctation of the aorta
DAo: Descending aorta
LV: Left ventricle/left ventricular
LVEF: Left ventricular ejection fraction
VO2_max_: Peak oxygen consumption
Re_DAo_: Reynolds number in the descending aorta
RV: Right ventricle/right ventricular
WSS: Wall shear stress
